# Second Primary Squamous Cell Carcinoma of the External Auditory Canal After Treatment of Nasopharyngeal Carcinoma: A Case Report

**DOI:** 10.7759/cureus.115450

**Published:** 2026-08-30

**Authors:** Lamyae Jabri, Btissam Amrani, Ahmed BenSghier, Soufiane Berhili, Mohamed Moukhlissi, Loubna Mezouar

**Affiliations:** 1 Radiation Oncology, Faculty of Medicine and Pharmacy of Oujda, Mohamed First University, Oujda, MAR; 2 Radiation Oncology, Centre Hospitalier Universitaire (CHU) Mohammed VI, Oujda, MAR; 3 Radiotherapy, Mohammed VI University Hospital, Oujda, MAR

**Keywords:** external auditory canal carcinoma, radiation oncology, second primary carcinoma, squamous cell carcinoma (scc), undifferentiated nasopharyngeal carcinoma

## Abstract

We describe a case of a 64-year-old man presenting with squamous cell carcinoma of the external auditory canal as a second primary malignancy after treatment of nasopharyngeal carcinoma, including conventional radiotherapy. Squamous cell carcinoma of the external auditory canal is a rare malignancy, with radiation-related cases even rarer. Management of this malignancy remains difficult due to the critical anatomical location and invasive nature, particularly in advanced stages. Several hypotheses have been advanced to explain the occurrence of second primary malignancies. We discuss this unusual malignancy, highlighting the diagnostic challenges encountered and the potential risk factors associated with second primary cancers.

## Introduction

External auditory canal carcinoma is a rare malignancy arising from the temporal bone [1], representing less than 0.2% of all head and neck cancers, with squamous cell carcinoma being the most common histological type [2]. It occurs mostly in people aged 50-60 years. Clinically, otorrhea is the most common presenting symptom; otalgia and hearing loss are also frequently observed [3]. Carcinoma of the external auditory canal frequently mimics benign and inflammatory ear conditions, leading to diagnostic challenges and often resulting in delayed detection [4]. The prognosis depends largely on the disease stage, with locally advanced tumors demonstrating more aggressive behavior and being associated with poorer outcomes [5]. Patients with a history of head and neck malignancies are at high risk for developing second primary cancers (SPCs), at a rate of 6.6% after nasopharyngeal carcinoma (NPC) treatment [6]. We present a case of squamous cell carcinoma in the EAC occurring after treatment of an NPC with induction chemotherapy and concurrent chemoradiotherapy. Herein, we discuss the case and provide a brief literature review.

## Case presentation

A 64-year-old male patient with a history of chronic smoking estimated at 35 pack-years was admitted for the management of an undifferentiated carcinoma of nasopharyngeal type (UCNT). The history of the disease dates back eight months prior to admission, with the onset of nasal obstruction and hearing loss. Following a nasopharyngeal biopsy and comprehensive staging workup, the tumor was classified as T3N2M0. The patient underwent induction chemotherapy with cisplatin and gemcitabine, of which he received three cycles. followed by concurrent chemoradiotherapy. The radiation fields of the external beam radiation included the nasopharynx, with the radiation delivered conventionally. A total radiation dose of 70 Gy (2 Gy/fr) was delivered. This was given concomitantly with cisplatin-based chemotherapy at a dose of 35 mg/m². The patient remained in good control. After 20 months, he presented with acute facial paralysis. A head and neck computed tomography scan was performed, suggesting tumor recurrence at the left lateral wall of the nasopharynx. Therefore, a nasopharyngoscopy with biopsy was done, showing no evidence of malignancy. The CT scan also showed chronic otitis. The patient remained under clinical surveillance. However, due to persistence of left facial paralysis and the onset of vertigo as well as otorrhea and otalgia, otoscopy was performed, revealing a whitish mass in the external auditory canal. A subsequent MRI of the nasopharynx demonstrated a lesion arising from the left ear, involving all ear structures with extension to the cerebellopontine angle (Figure 1)

**Figure 1:**
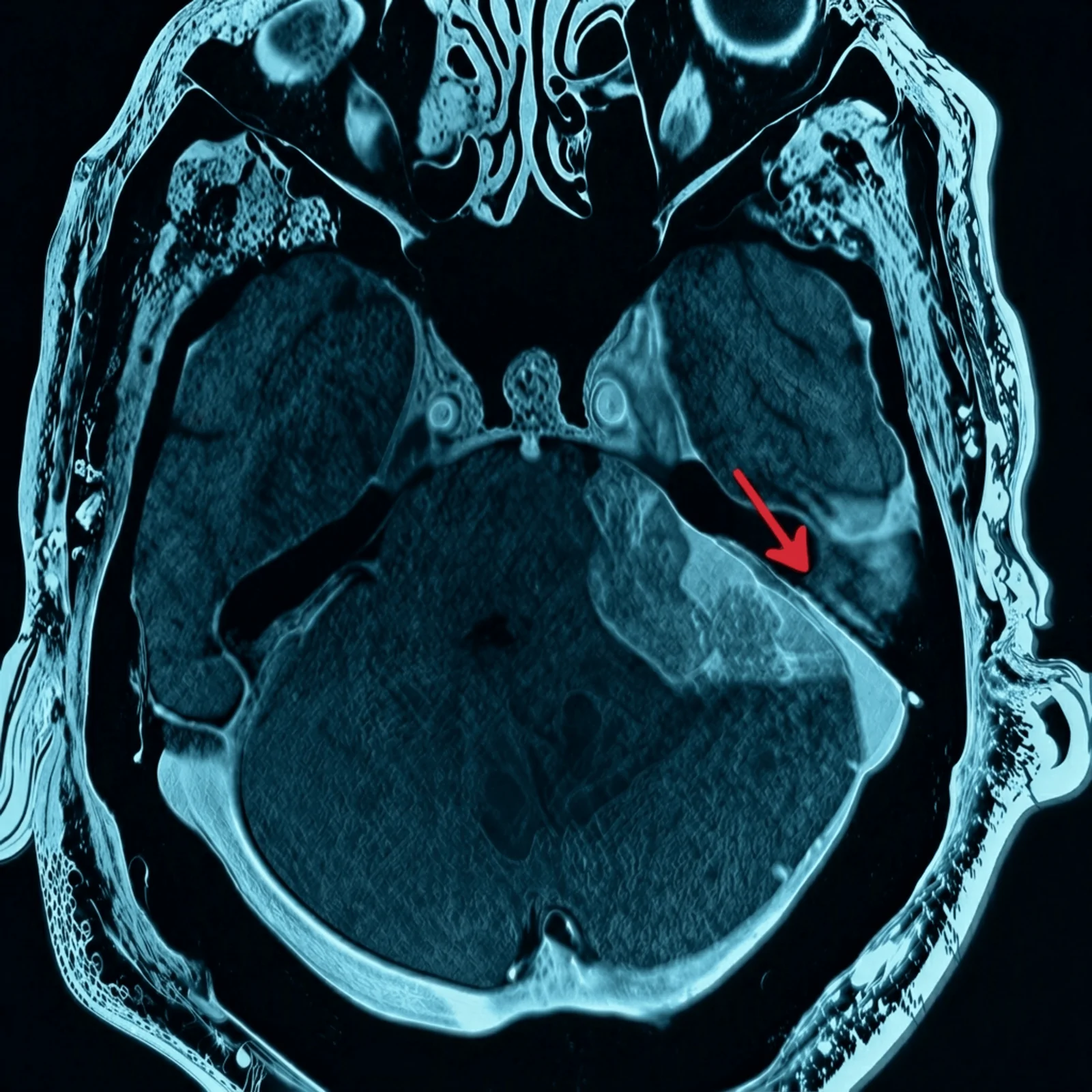
MRI in axial sequence T1 injected showing a lesion arising from the left ear with extension to the cerebellopontine angle

Biopsy findings were in favor of a well-differentiated infiltrating squamous cell carcinoma of the left external auditory canal (Figure 2).

**Figure 2:**
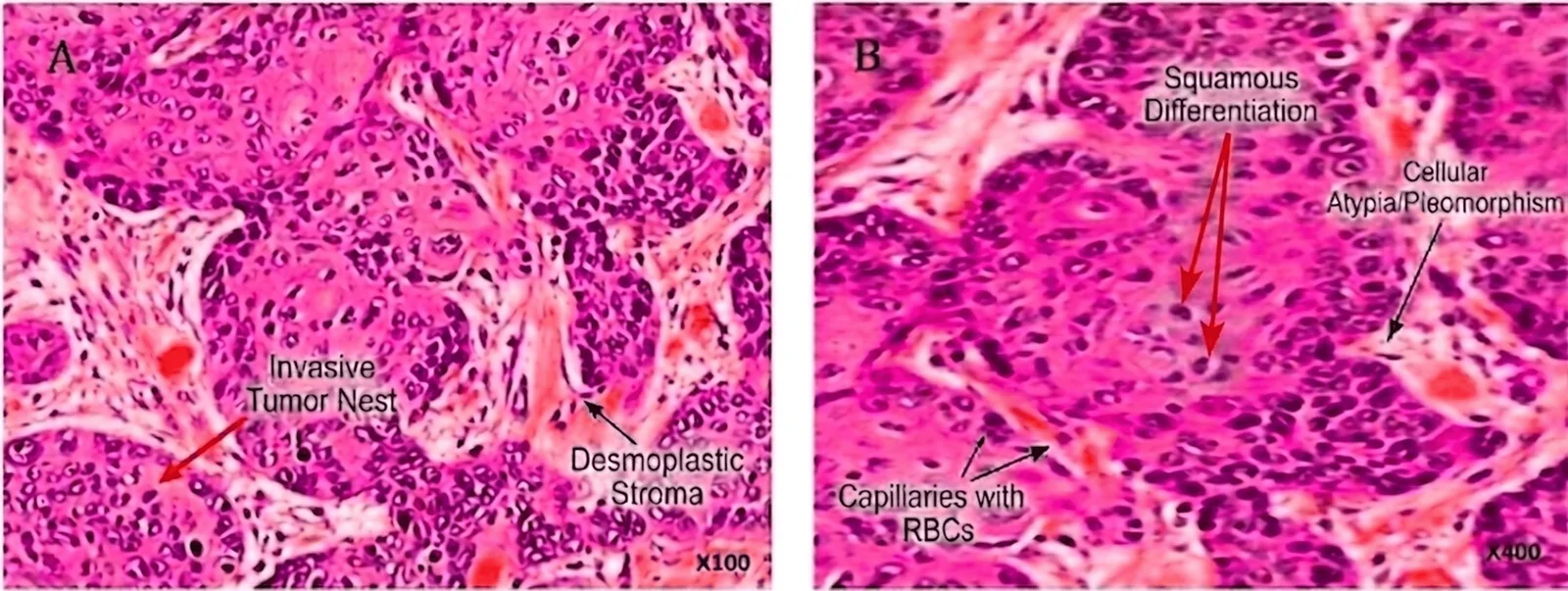
Tumor cells from the external auditory canal with multiple instances of cytonuclear atypia and mitosis A: Histopathological examination with H&E staining at ×100 magnification showing infiltrating tumor nests with central keratinization B: Histopathological examination with H&E staining at ×400 magnification showing cellular atypia and squamous differentiation Features are visually labeled.

Due to its extensive spread, the tumor was deemed unresectable. The patient was managed with palliative chemotherapy and intensity-modulated radiotherapy (IMRT) directed at the primary lesion, with field placement planned to minimize re-irradiation toxicity given the prior nasopharyngeal radiation site. Following treatment, the patient initially demonstrated clinical stabilization; however, subsequent follow-up revealed progressive deterioration marked by worsening neurological and otologic symptoms. He ultimately succumbed to the disease.

## Discussion

NPC is a relatively rare malignancy worldwide but remains highly prevalent in certain endemic regions, particularly in Asia. Incidence rates reach 20-30 per 100,000 in males and 7-13 per 100,000 in females in southeastern China, and approximately 20.2 and 7.8 per 100,000 in males and females, respectively, in Hong Kong [7]. Several risk factors have been identified, including environmental exposures [8] and genetic susceptibility [9], with Epstein-Barr virus infection recognized as the most significant etiological factor. NPC is a highly radiosensitive malignancy, and excellent locoregional control can be achieved with definitive chemoradiotherapy [10]. The earlier onset of NPC, combined with improved patient survival, has led to an increasing occurrence of SPCs, which may arise either as a consequence of initial treatment or from common etiological risk factors.

SPCs are defined as distinct malignancies that arise either synchronously or metachronously in an individual previously diagnosed with a different primary cancer [11]. Head and neck cancer survivors are at increased risk of developing an SPC. However, the incidence of SPC following NPC is variable and generally lower, as compared with other head and neck malignancies. A systematic review by Fanni Svärd et al., including 89,168 patients from 21 articles during the years 1961-2017, reported a mean rate of 6.6% [6].

Some studies have suggested that the risk of SPCs is elevated among patients diagnosed at a younger age (commonly defined as <40 years). This observation may be partly explained by the lower baseline incidence of cancer in younger patients, which results in a higher excess risk [7,12,13]. However, other studies reported a higher risk in older patients [14,15]. This finding aligns with the established pattern seen in primary malignancies, in which incidence is positively correlated with age.

The most common site of SPC is the oral cavity [7,16]. External auditory canal carcinoma has also been reported as an SPC in patients previously irradiated for NPC [17]. The occurrence of an SPC may be a result of adverse effects of cancer therapies; the main line of treatment of NPC is radiotherapy, which is known to be a risk factor for developing an SPC, and this causes DNA single-strand breaks, which can initiate senescence SPCs, telomere damage and shortening following chemotherapy or radiotherapy, and phenotypic and metabolic changes in stromal cells post-chemotherapy [11]. However, in our case, the latency period between the patient's first irradiation and the SPC was 20 months, which is shorter than the latency classically described for radiation-induced malignancies, which generally exceeds 5 years. This unusually early onset makes a direct causal relationship with prior radiotherapy unlikely.

Field cancerization, first described by Slaughter et al. [18], refers to the presence of genetically altered epithelial cells in the mucosa surrounding a primary tumor. This concept has been proposed to explain the occurrence of second primary malignancies, particularly in the head and neck region. According to this theory, chronic exposure to carcinogenic factors--most notably tobacco, which is also a recognized risk factor for external auditory canal carcinoma [4,1]--leads to the progressive accumulation of genetic alterations, ultimately resulting in the development of multiple independent malignant lesions.

Chronic ear infection and inflammation are well-recognized late complications in patients with NPC treated with radiotherapy [19]. Persistent inflammatory conditions, particularly chronic otitis media, as observed in our case, have been reported as potential risk factors for the development of squamous cell carcinoma [4,20]. This association is thought to be mediated by long-standing inflammatory processes that promote epithelial damage, increased cellular turnover, and subsequent metaplastic or dysplastic changes, ultimately predisposing to malignant transformation [1].

Patients with primary head and neck malignancies are at increased risk of developing second primary tumors. Schwartz et al. [21] reported a 19% incidence, with 41% classified as synchronous and 59% as metachronous. Therefore, the possibility of a previously undetected synchronous lesion cannot be excluded, particularly given the relatively short interval between the treatment of NPC and subsequent tumor detection.

## Conclusions

In summary, we report a rare and aggressive tumor arising as a second primary cancer in a patient previously treated with radiotherapy for nasopharyngeal carcinoma. In this case, chronic ear inflammation developing after the treatment of the initial malignancy may have contributed to the development of external auditory canal carcinoma, although the possibility of an initially undetected synchronous malignancy cannot be excluded. This observation highlights the importance of close and sustained surveillance, even in the early post-treatment period, particularly in patients presenting with persistent or chronic inflammatory ear conditions.
